# Oxycodone for analgesia in children undergoing endoscopic retrograde cholangiopancreatography: a randomized, double-blind, parallel study

**DOI:** 10.3389/fphar.2024.1515501

**Published:** 2025-01-08

**Authors:** Wei Ji, Liping Sun, Yue Huang, Jie Bai, Jijian Zheng, Kan Zhang

**Affiliations:** ^1^ Department of Anesthesiology and Pediatric Clinical Pharmacology Laboratory, Shanghai Children’s Medical Center, Affiliated to Shanghai Jiao Tong University School of Medicine, Shanghai, China; ^2^ National Children’s Medical Center, Shanghai, China

**Keywords:** oxycodone, endoscopic retrograde cholangiopancreatography (ERCP), visceral pain, children, inflammation

## Abstract

**Background:**

Postoperative visceral pain is a common complication after endoscopic retrograde cholangiopancreatography (ERCP). In this study, we compared the analgesic and anti-inflammatory effects of oxycodone and fentanyl in children undergoing ERCP.

**Methods:**

A single-center, randomized, double-blind study was conducted at a tertiary care hospital affiliated with Shanghai Jiao Tong University. Eighty-two pediatric patients aged 2–18 years who were scheduled for elective ERCP were randomly assigned to receive either oxycodone (0.2 mg/kg) or fentanyl (2 μg/kg). The postoperative pain was evaluated after 10 min, 20 min, and 30 min in the post-anesthesia care unit (PACU) as well as 6 h and 24 h in the ward following ERCP. Additionally, inflammatory cytokines in the serum, including tumor necrosis factor (TNF)-α, interleukin (IL)-6, and IL-10 were examined by blood sampling at baseline, 6 h, and 24 h after ERCP.

**Results:**

Compared to fentanyl, children receiving oxycodone had significantly lower pain scores at 30 min, 6 h, and 24 h after ERCP, while the scores at 10 and 20 min were similar in both groups. We also found that fewer patients had pain scores ≥3 at 6 h and 24 h after the procedure in the oxycodone group [36.6% (15/41) vs. 61.0% (25/41) at 6 h, 34.1% (14/41) vs. 58.5% (24/41) at 24 h, *p* = 0.027 for both cases]. Furthermore, fewer children in the oxycodone group had elevated inflammatory cytokines (IL-6 at 6 h and TNF-α at 24 h after ERCP) compared to the fentanyl group. The incidence of postoperative vomiting was also lower among children receiving oxycodone [14.1% (7/41) vs. 24.4% (10/41), *p* = 0.032].

**Conclusion:**

Oxycodone (0.2 mg kg^−1^) can provide effective analgesia and stable hemodynamics in children undergoing ERCP. This analgesic characteristic may be related to amelioration of inflammation after ERCP.

**Clinical Trial Registration:**

www.chictr.org.cn, identifier ChiCTR2300074473.

## Introduction

Endoscopic retrograde cholangiopancreatography (ERCP) is a valuable diagnostic and therapeutic procedure for managing complex hepatopancreatobiliary diseases in children. Although ERCP is minimally invasive, post-ERCP pain remains a frequent and distressing complication that affects 7%–28% of pediatric patients (Chen et al., 2022). Such post-ERCP pain can be attributed to multiple factors, including systematic inflammation, sphincter of Oddi dysfunction, and complications like post-ERCP pancreatitis (PEP) (Maple et al., 2009; Thompson, 2001).

The management of post-ERCP pain in children remains a challenge owing to the inconsistent guidelines among experts and lack of consensus on the most effective analgesic strategies (Chen et al., 2022; Cai et al., 2021; Vittinghoff et al., 2018, 2024). Current pharmacological approaches typically involve the use of opioids or non-steroidal anti-inflammatory drugs (NSAIDs), which have their own limitations (Macintyre et al., 2022). Fentanyl is a potent opioid that is frequently used in the treatment of moderate-to-severe pain, including pediatric post-ERCP pain, but it is particularly effective for acute intraoperative and postoperative incisional pain. However, like other μ-opioid agonists, fentanyl is associated with adverse events, such as respiratory depression and vomiting (Hill et al., 2020; Wang et al., 2022). Moreover, there are concerns that opioids may increase the risk of acute pancreatitis owing to sphincter of Oddi dysfunction (Singh, 2020). Although NSAIDs have been widely used in adults for post-ERCP pain and demonstrated efficacy in reducing inflammation, evidence supporting their use in children undergoing ERCP is limited, with the key considerations being dose adjustment in children, optimal administration route (oral, rectal, or systemic), timing of administration (before or after the procedure), and NSAID-related potential adverse events like hepatic or renal function impairment and bleeding risks (Brasher et al., 2014). Given these limitations, exploring alternative analgesics beyond μ-opioids and NSAIDs is warranted.

Oxycodone is a selective agonist of both μ- and κ-opioid receptors, with primary actions on both central and peripheral κ-opioid receptors as well as lower affinity to the μ-opioid receptor (Marie and Noble, 2023), which allows better control over visceral pain with fewer μ-opioid-related adverse events (Arendt-Nielsen et al., 2009; Larsson et al., 2008; Joshi et al., 2000). Animal experiments and clinical studies have further suggested that oxycodone may ameliorate inflammatory responses in pathological conditions like inflammatory bowel diseases and joint inflammation (Szczepaniak et al., 2022; Salaga et al., 2017). Additionally, activation of the κ-opioid receptor of the immune cells in the central nervous system and peripheral blood (e.g., microglia, macrophages, and B and T lymphocytes) could modulate the balance between pro- and anti-inflammatory activities (Szczepaniak et al., 2022; Salaga et al., 2017; Machelska and Celik, 2020). Therefore, we speculate that oxycodone may provide superior analgesic and anti-inflammatory effects in children undergoing ERCP compared to other drugs, which remains to be clarified.

The present study was primarily an investigation of whether oxycodone provides better pain relief than fentanyl in pediatric ERCP patients. Additionally, we explored whether oxycodone reduces the inflammatory cytokines in the serum.

## Materials and methods

### Pediatric patients

The prospective study was approved by the Institutional Review Board of Shanghai Children’s Medical Center (No. SCMCIRB-K2022186-1) and was registered with the Chinese Clinical Trial Registry (www.chictr.org.cn, No. ChiCTR2300074473). This study conformed to all the guidelines of the Declaration of Helsinki (World Medical, 2024). Written informed consent was obtained from the parents or legal guardians of all children aged 2–18 years. Meanwhile, written informed consent was also obtained from children aged ≥8 years.

All children who underwent ERCP at Shanghai Children’s Medical Center from September to December 2023 were enrolled in this study and allocated randomly into the treatment (receiving oxycodone) and control (receiving fentanyl) groups. The inclusion criteria were as follows: 1) children aged 2–18 years; 2) children suffering from pancreatic and biliary tract diseases that required ERCP; 3) children designated grades 1–2 based on the American Society of Anesthesiologists—Physical Status (ASA-PS). Children were excluded if they had one or more of the following conditions: 1) history of allergy to the experimental medication; 2) active asthma within the past 6 months; 3) severe cardiovascular, hepatic, or renal dysfunction; 4) neurological and psychiatric disorders; 5) chronic pain or had received opioids within the previous 24 h; 6) patients or guardians refusing participation in the study.

### Anesthetic procedures

All pediatric patients fasted for 6 h avoided clear fluids for at least 2 h before the surgery, and received an intravenous catheter in the ward. Upon entering the ERCP operation theater, the electrocardiogram, non-invasive blood pressure, pulse oximetry, and bispectral index were monitored routinely. The induction drugs and their administration sequence were the same for both groups: midazolam (0.1 mg/kg), propofol (3 mg/kg), oxycodone or fentanyl, and rocuronium (0.6 mg/kg). Considering equianalgesic opioid doses, we standardized the fentanyl and oxycodone doses in terms of morphine milligram equivalents (10 mg of oxycodone = 100 μg of fentanyl = 10 mg of morphine) (Gordon et al., 1999; Aubrun et al., 2019). Therefore, oxycodone (0.2 mg/kg, Jiangsu Nhwa Pharmaceutical Co., Ltd., China) or fentanyl (2 μg/kg, Hubei Humanwell Group Co., Ltd., China) was used. Two minutes following induction, endotracheal intubation was performed by a senior attending anesthesiologist. Pressure-controlled ventilation was used with a tidal volume of 6–10 mL/kg and age-adjusted frequency to maintain end-tidal carbon dioxide levels between 35 and 45 mmHg. Sevoflurane was inhaled to maintain the bispectral index between 40 and 60. If the blood pressure increased by more than 20% above baseline, oxycodone or fentanyl at half of the induction dose was administered. After extubation at the end of surgery, all children were admitted to the post-anesthesia care unit (PACU) for monitoring and were transferred to the ward once the discharge criteria were met (modified Aldrete score ≥9 points).

### Assessment and management of pain

One anesthesiologist evaluated all-cause pain after ERCP (PEP or non-PEP) at five timepoints as follows: 10 min, 20 min, and 30 min after the children arrived at the PACU; 6 h and 24 h after discharge from the PACU to the ward. Based on the pediatric ages of the patients, two pain scales were used. For children aged 2–7 years, the face, legs, activity, cry and consolability (FLACC) scale with a maximum score of 10 points comprising five aspects was used; for children older than 7 years, the numerical rating scale (NRS) with a straight line divided into 10 segments from 0 (“no pain”) to 10 (“the worst imaginable pain”) was used. Rescue analgesia was administered by the recovery nurse according to clinical need.

The incidence of perioperative adverse events was monitored and recorded, including laryngospasm, oxygen desaturation (<90%), vomiting, delayed emergence, and PEP. The duration of the surgery was defined as the time from disinfection to the end of ERCP. The extubation time was defined as the time from discontinuation of sevoflurane to removal of the endotracheal tube. PEP was diagnosed internally by a doctor based on a series of clinical symptoms within 72 h after the procedure (e.g., serum amylase and lipase levels exceeding three times the upper limits of normal values along with visceral pain).

Additionally, inflammatory cytokines in the serum, including the human tumor necrosis factor (TNF)-α and interleukin (IL)-6, and IL-10, were measured at three time points (before procedure, 6 h and 24 h after procedure). Immunoassays from Invitrogen (Thermo Fisher Scientific Inc., Waltham, MA, United States) were used for the measurements according to manufacturer instructions. In our hospital, the laboratory reference values were 5.4 pg/mL for IL-6, 12.9 pg/mL for IL-10, and 16.5 pg/mL for TNF-α, with the limit of detection (LOD) for IL-6, IL-10, and TNF-α being 2.44 pg/mL. Considering that concentrations below the LOD were labeled as non-detectable, we transformed the continuous variables into ordinal categorical variables, namely, non-detectable (concentrations below the LOD), below the reference value (concentrations between the LOD and laboratory reference values), and above the reference value.

### Blind and randomization

The children were allocated into groups receiving oxycodone or fentanyl by a simple digital randomization method, with group allocation and patient numbers in sealed envelopes. One nurse who was not involved in the study prepared the drugs for anesthesia. One attending anesthesiologist who was blinded to the allocation was responsible for evaluating pain and recording data.

### Sample size

Based on the primary endpoint of our pilot study, the average pain scores 30 min after ERCP were 2 for the oxycodone group and 3 for the fentanyl group. We assumed that the effect size of the pain scores 30 min after surgery was 1. The required sample size was calculated as 39 patients for each group by assuming that the type I error was 0.05 (two-sided) and power was 80%. Taking into account a 15% dropout rate, the total sample size required was 90 patients.

### Data analysis

The normality of distribution was examined using the Shapiro–Wilk test. Continuous variables were shown as mean ± standard deviation (SD) if they were distributed normally or as median with interquartile range (IQR) otherwise. The numerical variables were compared using the Student’s t-test or Mann–Whitney U test. The Hodges–Lehmann method was used to determine the median values and corresponding 95% confidence intervals (CIs) of the intergroup differences in pain scores. The categorical variables were expressed as numbers (%) and compared using the χ^2^ or Fisher χ^2^ test. The continuous variables (heart rate (HR), mean blood pressure (MBP)) between the groups were compared using repeated measures analysis of variance (ANOVA). For the pain scores, a Kruskal–Wallis one-way ANOVA with multiple comparisons within the groups was performed. The statistical analyses were performed using IBM SPSS Statistics 26.0 (IBM Corp., Armonk, NY, United States) and GraphPad Prism 9.3 (GraphPad Software Inc, San Diego, CA). A *p*-value < 0.05 was considered to be statistically significant.

## Results

**FIGURE 1:**
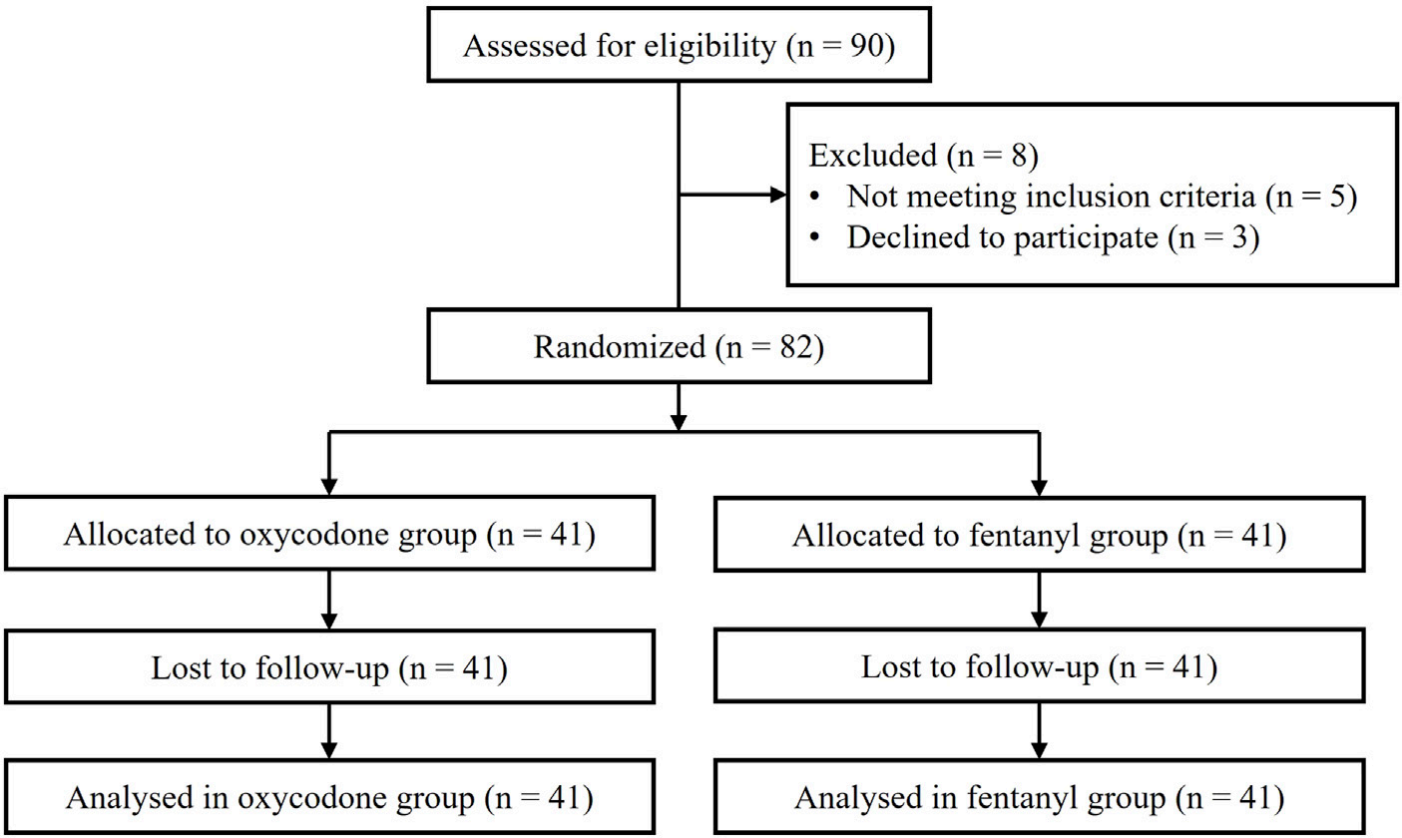
Flow diagram showing the numbers of individuals at each stage of the study.

All patient data were collected between December 2022 and November 2023. Of the total of 90 pediatric patients recruited initially, 82 were allocated randomly into the two groups, while 8 children did not meet the inclusion criteria. Finally, the 82 patients received the analgesic agents (oxycodone or fentanyl) and completed the follow-up. The CONSORT flow diagram is shown in Figure 1. The demographic data (age, sex, height, and weight), operation time, and extubation time were not significantly different between the two groups (all *p* > 0.05, Table 1). In addition, the inflammatory cytokine levels at baseline were comparable between the groups (Supplementary Tables S1–S3).

**TABLE 1:** Patient demographic and perioperative data.

	Oxycodone (*n* = 41)	Fentanyl (*n* = 41)	*p*-value
Age, year	8.2 ± 3.8	9.3 ± 3.9	0.177
Age, *n* (%)			
2 ≤ age < 7 years	18 (43.9)	13 (31.7)	0.478
7 ≤ age < 18 years	23 (56.1)	28 (68.3)	
Gender, *n* (%)			
Male	18 (43.9)	15 (36.6)	0.425
Female	23 (56.1)	26 (63.4)	
Height, cm	126.5 ± 24.4	133.8 ± 24.3	0.263
Weight, kg	27.2 ± 13.6	30.7 ± 14.2	0.177
ASA-PS			
Ⅰ	37 (90.2)	36 (87.8)	0.724
Ⅱ	4 (9.8)	5 (12.2)	
Morphine milligram equivalent, mg	5.4 ± 2.7	6.1 ± 2.8	0.263
Procedure time, min	40.6 ± 11.3	36.3 ± 9.4	0.067
Extubation time, min	7.4 ± 1.6	7.4 ± 1.4	0.885

Data are shown as mean ± standard deviation or *n* (%). A *p*-value < 0.05 was considered to be statistically significant.

**FIGURE 2:**
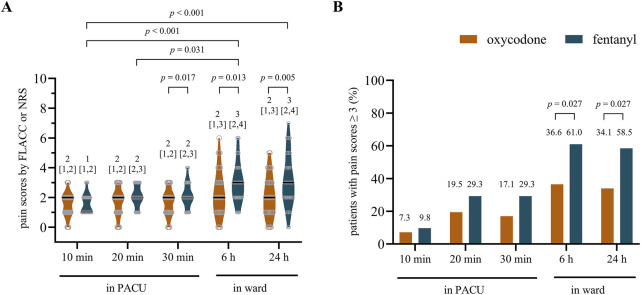
**(A)** Postprocedural pain scores and **(B)** proportions of patients with pain scores ≥3 in the two groups. A *p*-value < 0.05 was considered significantly different between the groups. The data are shown as median with interquartile range (IQR).

In this study, the morphine milligram equivalents of oxycodone and fentanyl used for analgesia were similar (5.4 ± 2.7 mg of oxycodone vs. 6.1 ± 2.8 mg of fentanyl, *p* = 0.263). The pain scores were similar at 10 min and 20 min post-ERCP in patients of both groups [median (IQR): 2 (Chen et al., 2022; Maple et al., 2009) vs. 1 (Chen et al., 2022; Maple et al., 2009) with effect size (95% CI), 0 (0, 0), *p* = 0.870 at 10 min; 2 (Chen et al., 2022; Maple et al., 2009) vs. 2 (Maple et al., 2009; Thompson, 2001) with effect size (95% CI), 0 (0, 0), *p* = 0.062 at 20 min)]. However, patients in the oxycodone group showed less pain intensity than those in the fentanyl group from 30 min to 24 h after surgery [2 (Chen et al., 2022; Maple et al., 2009) vs. 2 (Maple et al., 2009; Thompson, 2001) with effect size (95% CI), 0 (−1, 0), *p* = 0.017 at 30 min; 2 (Chen et al., 2022; Thompson, 2001) vs. 3 (Maple et al., 2009; Cai et al., 2021) with effect size (95% CI), −1 (−1, 0), *p* = 0.013 at 6 h; 2 (Chen et al., 2022; Thompson, 2001) vs. 3 (Maple et al., 2009; Cai et al., 2021) with effect size (95% CI), −1 (−2, 0), *p* = 0.005 at 24 h; Figure 2A]. Additionally, we found that the numbers of patients with pain scores ≥3 at 6 h and 24 h were significantly higher in the fentanyl group than the oxycodone group [36.6% (15/41) vs. 61.0% (25/41) at 6 h, 34.1% (14/41) vs. 58.5% (24/41) at 24 h, both *p* = 0.027, Figure 2B]. These results indicate that oxycodone has better and long-lasting analgesic effects than fentanyl. There were no differences in the HRs and MBPs during ERCP between the two groups (all *p* > 0.05, Table 2).

**TABLE 2:** Patient hemodynamics during the ERCP procedure.

	At baseline	Endotracheal intubation	Endoscope insertion	At the end of surgery	*p*-value
HR					
Oxycodone	101 ± 13	102 ± 14	99 ± 12	99 ± 11	0.056
Fentanyl	109 ± 14	104 ± 14	104 ± 14	103 ± 13	
MBP					
Oxycodone	65 ± 4	68 ± 4	70 ± 5	65 ± 4	0.432
Fentanyl	64 ± 5	68 ± 5	71 ± 5	64 ± 4	

Data are shown as mean ± standard deviation. A *p*-value < 0.05 was considered to be statistically significant between the groups. HR, heart rate; MBP, mean blood pressure.

**FIGURE 3:**
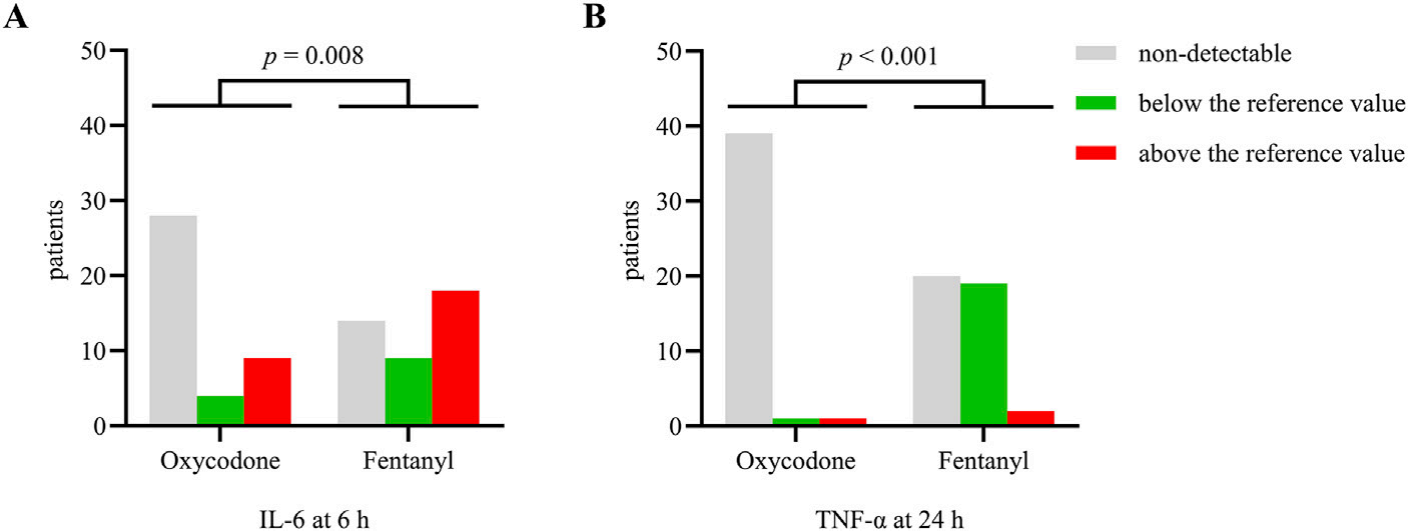
Numbers of patients with different levels of inflammatory cytokines **(A)** IL-6 at 6 h and **(B)** TNF-α at 24 h after ERCP. The data are expressed as *n*(%). A *p*-value < 0.05 was considered significantly different between the groups.

Post-ERCP pain is known to be strongly associated with increased release of pro-inflammatory cytokines. We further tested the levels of inflammatory cytokines (IL-6, IL-10, and TNF-α) through blood sampling at baseline, 6 h, and 24 h after the procedure (Supplementary Tables S1–S3). The distributions of patients with inflammatory cytokines differed between the groups. At 6 h after ERCP, more patients in the fentanyl group had IL-6 levels above the reference value than the oxycodone group. At 24 h after ERCP, more children in the fentanyl group had elevated TNF-α levels than in the oxycodone group, although the concentrations were still below the reference value (Figure 3). The changes in the levels of anti-inflammatory cytokine IL-10 before and after ERCP were similar in both groups. These results suggest that patients receiving oxycodone experience fewer inflammatory responses than those receiving fentanyl.

To examine the safety of the analgesic agents, we analyzed the incidences of perioperative and postoperative adverse events. The incidence of postoperative vomiting was lower in children receiving oxycodone [14.1% (7/41) vs. 24.4% (10/41), *p* = 0.032], while the incidence of PEP was similar between the groups [7.3% (3/41) vs. 9.8% (4/41), *p* = 0.693]. Desaturation, laryngospasm, and delayed emergence did not occur in either group.

## Discussion

A prospective randomized controlled study was conducted to evaluate whether oxycodone could provide better pain relief for pediatric post-ERCP patients compared to fentanyl. Our study revealed that oxycodone not only provides better pain relief with lower post-ERCP pain scores than fentanyl but also exhibits long-lasting analgesic effects in pediatric patients undergoing ERCP. Furthermore, our study revealed that the analgesic effects of oxycodone may be related to promising anti-inflammatory responses; these advantages of oxycodone were also observed to not cause more adverse events.

At present, oxycodone shows better and longer-lasting analgesic effects in children undergoing ERCP. Similar to the findings of our study, oxycodone has demonstrated better visceral analgesia than μ-opioids, such as morphine or fentanyl derivatives, in adults undergoing abdominal laparoscopic surgeries (Yang et al., 2024; Liu et al., 2021; Lenz et al., 2009). Until now, very few studies have reported the efficacy of oxycodone for analgesia in ERCP procedures. The analgesic effects of oxycodone are likely mediated through both central and peripheral κ-receptors, which have been suggested to be involved in visceral pain (Arendt-Nielsen et al., 2009; Larsson et al., 2008; Joshi et al., 2000). The longer duration of action of oxycodone may be related to its pharmacokinetics and active metabolism. The duration of action of oxycodone is typically 3–4 h, while the effects of fentanyl typically last for 1 h (Olkkola et al., 2013; Ziesenitz et al., 2018).

Notably, we found that oxycodone has a promising role in reducing inflammatory pain compared to fentanyl, along with temporal changes in the earlier increase in IL-6 and later increase in TNF-α levels. In addition to mechanical stimulus (dilation and contraction), pro-inflammatory cytokines (IL-6 and TNF-α) have been shown to mediate visceral pain (Chen et al., 2022; Kivell and Prisinzano, 2010). The treatment efficacy is often reflected in the levels of pro-inflammatory cytokines. The study by An et al. (2019) showed that oxycodone (0.1 mg/kg) reduced serum TNF-α levels and pain scores more effectively than sufentanil in the period from 6 h to 24 h after laparoscopic cholecystectomy. These findings are consistent with our results, which show lower incidence of visceral pain with oxycodone.

Compared to traditional μ-opioid receptors, oxycodone may mediate analgesic and anti-inflammatory effects by modulating the functions of the immune cells in the peripheral tissues as well as the central nervous system from pro-inflammatory to anti-inflammatory states while reducing the release of pro-inflammatory cytokines derived from immune cells (Szczepaniak et al., 2022; Machelska and Celik, 2020). Unlike NSAIDs that primarily inhibit cyclooxygenase (COX) enzymes, oxycodone can address pain types that are resistant to NSAIDs (e.g., severe pain), preserve platelet functions, and minimize the risk of gastrointestinal damage (Brasher et al., 2014). These characteristics make oxycodone a particularly practical and promising option for use in pediatric ERCP.

Consistent with previous research, oxycodone provides stable hemodynamics (Guo et al., 2021). We also did not observe any opioid-related respiratory depression and oxygen desaturation during ERCP, which may be related to the choice of anesthesia. In fact, all children were mechanically ventilated via endotracheal tube, unlike adults, who underwent propofol-based sedation with autonomous respiration (Guo et al., 2021; Liu et al., 2020; Fassoulaki et al., 2015). This highlights the clinical safety of oxycodone in children when an endotracheal tube is used. We also observed lower incidence of post-ERCP vomiting in the oxycodone group compared to the fentanyl group, suggesting that oxycodone is well tolerated and causes less discomfort in children.

Our study has several limitations. First, owing to the lower incidence of PEP, all-cause post-ERCP pain (PEP and non-PEP) was reported in the current study. Although the US Food and Drug Administration has approved extended-release oxycodone for children with severe pain (Yang et al., 2016), the analgesic effects of oxycodone for pediatric visceral pain induced by pancreatitis still needs clarification (Yang et al., 2016). Second, the analgesic effects of oxycodone were observed within the first 24 h after surgery. However, an earlier retrospective study showed that post-ERCP pain tends to peak within 24 h (Chen et al., 2022), so it is unknown whether oxycodone continues to provide better analgesic effects beyond 24 h after ERCP. Extended follow-up is therefore needed in future research. Third, the present study was conducted at a single tertiary care hospital, where patients with more severe conditions may be overrepresented compared to those treated in community clinics. As a result, these findings may not be generalizable beyond this single-center setting. Future studies should include multicenter trials to address this limitation. Fourth, the oxycodone used in this study showed reduced pro-inflammatory cytokinesis; it is emphasized here that oxycodone is not a first-line anti-inflammatory medication like an NSAID and that it likely reduces inflammation indirectly by alleviating pain.

## Conclusion

Oxycodone treatment at 0.2 mg/kg is shown to provide effective analgesia and stable hemodynamics in children undergoing ERCP. This analgesic effect may also be related to the amelioration of inflammation after ERCP.

## Data Availability

The raw data supporting the conclusions of this article will be made available by the authors without undue reservation.
