# Supplementary material for: Oxycodone for analgesia in children undergoing endoscopic retrograde cholangiopancreatography: a randomized, double-blind, parallel study
Source: Front Pharmacol. 2025 Jan 8;15:1515501. doi: 10.3389/fphar.2024.1515501 (PMC11751031; doi:10.3389/fphar.2024.1515501)
Supplement: Supplementary file 1 [file Table1.docx]

Table S1 IL-6 measured at baseline, 6 h and 24 h after ERCP

|  | oxycodone  (n = 41) | fentanyl  (n = 41) | *p* value |
| --- | --- | --- | --- |
| at baseline |  |  |  |
| non-detectable | 34 (82.9) | 34 (82.9) | 0.867 |
| below the reference value | 4 (9.8) | 3 (7.3) |  |
| above the reference value | 3 (7.3) | 4 (9.8) |  |
|  |  |  |  |
| at 6 h |  |  |  |
| non-detectable | 28 (68.3) | 14 (34.1) | 0.008 |
| below the reference value | 4 (9.8) | 9 (22.0) |  |
| above the reference value | 9 (22.0) | 18 (43.9) |  |
|  |  |  |  |
| at 24 h |  |  |  |
| non-detectable | 20 (48.8) | 12 (29.3) | 0.080 |
| below the reference value | 12 (29.3) | 11 (26.8) |  |
| above the reference value | 9 (22.0) | 8 (43.9) |  |

The reference value of IL-6 was 5.4 pg ml^-1^. Data were expressed as n (%). *p* value <0.05 was considered significantly different.

Table S2 TNFα measured at baseline, 6 h and 24 h after ERCP

|  | oxycodone  (n = 41) | fentanyl  (n = 41) | *p* value |
| --- | --- | --- | --- |
| at baseline |  |  |  |
| non-detectable | 36 (87.8) | 39 (95.1) | 0.383 |
| below the reference value | 4 (9.8) | 1 (2.4) |  |
| above the reference value | 1 (2.4) | 1 (2.4) |  |
|  |  |  |  |
| at 6 h |  |  |  |
| non-detectable | 32 (78.0) | 32 (78.0) | 1.0 |
| below the reference value | 8 (19.5) | 8 (19.5) |  |
| above the reference value | 1 (2.4) | 1 (2.4) |  |
|  |  |  |  |
| at 24 h |  |  |  |
| non-detectable | 39 (95.1) | 20 (48.8) | < 0.001 |
| below the reference value | 1 (2.4) | 19 (46.3) |  |
| above the reference value | 1 (2.4) | 2 (4.9) |  |

The reference value of TNFα was 16.5 pg ml^-1^. Data were expressed as n (%). *p* value <0.05 was considered significantly different.

Table S3 IL-10 measured at baseline, 6 h and 24 h after ERCP

|  | oxycodone  (n = 41) | fentanyl  (n = 41) | *p* value |
| --- | --- | --- | --- |
| at baseline |  |  |  |
| non-detectable | 37 (90.2) | 40 (97.6) | 0.359 |
| below the reference value | 4 (9.8) | 1 (2.4) |  |
| above the reference value | 0 | 0 |  |
|  |  |  |  |
| at 6 h |  |  |  |
| non-detectable | 37 (90.2) | 40 (97.6) | 0.359 |
| below the reference value | 4 (9.8) | 1 (2.4) |  |
| above the reference value | 0 | 0 |  |
|  |  |  |  |
| at 24 h |  |  |  |
| non-detectable | 36 (87.8) | 39 (95.1) | 0.383 |
| below the reference value | 4 (9.8) | 1 (2.4) |  |
| above the reference value | 1 (2.4) | 1 (2.4) |  |

The reference value of IL-10 was 12.9 pg ml^-1^. Data were expressed as n (%). *p* value <0.05 was considered significantly different.
